# Understanding rice production stagnation in the Philippines: Regional evidence and development implications

**DOI:** 10.1371/journal.pone.0335344

**Published:** 2025-10-24

**Authors:** Henry A. Bartelet, Alenn Jhulia D. Prodigalidad, Janelle S. Dy, Jan Gabriel N. Manzano

**Affiliations:** 1 John Gokongwei School of Management, Ateneo de Manila University, Quezon City, National Capital Region, Philippines; 2 School of Project Management, Faculty of Engineering, The University of Sydney, Sydney, New South Wales, Australia; 3 Department of Environmental Science, Ateneo de Manila University, Quezon City, National Capital Region, Philippines; Lusofona University of Humanities and Technologies: Universidade Lusofona de Humanidades e Tecnologias, PORTUGAL

## Abstract

Despite decades of investment in agricultural research and rice development, the Philippines continues to face stagnation in domestic rice production. This persistent trend, occurring despite a sizable arable land base and large rural workforce, has contributed to the country’s growing reliance on rice imports to meet national food needs. In response, the government has implemented a tariff-based import system designed to fund domestic productivity improvements. While previous studies have explored the technical and environmental constraints affecting rice production, such as irrigation shortfalls, land degradation, and climate variability, few have examined recent national trends using regionally disaggregated data alongside qualitative insights. This study fills that gap by analysing official production statistics from 2013 to 2023 across all regions of the Philippines, complemented by contextual data from farming communities and policy reviews. The results reveal that widespread stagnation is primarily driven by limits to both land expansion and yield growth. However, a handful of regions have significantly increased output in recent years, largely due to targeted public investment in irrigation, improved seed technologies, and institutional support. These cases offer important lessons for policy, while also underscoring the risks posed by climate shocks and competing land uses. By identifying both barriers and enablers of rice sector performance, this research contributes to more regionally responsive and inclusive policy strategies aimed at strengthening food security, rural livelihoods, and agricultural resilience in the face of evolving development challenges.

## Introduction

Rice remains the cornerstone of food security, cultural identity, and rural livelihoods across much of Asia, especially in countries where it constitutes the primary dietary staple [1,2]. In the Philippines, rice is not only a vital food source but also a political commodity and a key driver of agricultural employment. Ensuring a stable domestic supply is therefore critical to household food access, farmer incomes, and broader rural development outcomes. Yet despite decades of investment in rice science, infrastructure, and policy reform, the country continues to struggle with stagnant domestic production. With a population of over 115 million in 2023 [3], the Philippines faces rising consumption needs that increasingly outpace domestic supply. In response, the country has turned to rice imports as a buffer, a move that helps meet short-term demand but also raises concerns about food sovereignty, exposure to international price shocks, and the long-term viability of the domestic rice sector [4,5]. These dynamics reflect deeper structural challenges facing food systems in many lower-middle income countries, where the intersection of land use pressures, climate vulnerability, and uneven state support continues to shape agricultural production trajectories and rural livelihoods [6,7].

Rice has long been central not only to Filipino diets but also to rural livelihoods and national identity. For decades, the Philippine government maintained a protectionist stance on rice markets, using quantitative import restrictions (QRs) to shield local producers from cheaper foreign rice. Originally implemented during the Marcos regime, this policy sought to ensure national self-sufficiency and safeguard the incomes of smallholder farmers. However, in 2019, the Rice Tariffication Law (Republic Act No. 11203) marked a pivotal shift in the country’s rice policy landscape. In compliance with long-standing World Trade Organization (WTO) commitments, the law replaced import quotas with a tariff-based regime, liberalizing rice imports while creating a fund (the Rice Competitiveness Enhancement Fund, RCEF) to support domestic producers through investment in seeds, mechanization, and extension services. Proponents of the Rice Tariffication Law (RTL) argued that liberalizing rice imports would enhance market transparency, reduce consumer prices, and address inefficiencies in domestic rice supply chains [8,9].

While initial analyses suggest that lower prices have delivered short-term gains for consumers, particularly among poor households, debate continues over the broader developmental impact of the reform [10]. In many rice-producing regions, smallholder farmers now face heightened market vulnerability, especially where public investment in infrastructure, technology, and extension services remains limited. These dynamics reflect a deeper tension confronting many lower and middle-income countries on how to align global trade commitments with inclusive, climate-resilient agricultural development. Although the Rice Competitiveness Enhancement Fund (RCEF) aims to mitigate negative impacts by supporting domestic producers, questions remain about whether it adequately addresses the structural barriers behind stagnating rice production. Beyond short-term price adjustments and input subsidies, more comprehensive and regionally differentiated interventions may be needed to revitalize the sector. A key policy question thus remains whether the RTL, and compensatory measures like the RCEF, can meaningfully address the structural drivers of stagnating domestic rice production.

The stagnation of rice production in the Philippines has been the subject of considerable research over the past decades, with studies identifying a range of biophysical, economic, and policy-related factors. Early analyses highlighted land conversion driven by urbanization and industrial expansion as a major constraint, with rice farmland declining from 4 million hectares in 1991 to 3.8 million hectares by 2002 [11]. Others have examined the impacts of natural disasters [12], soil degradation [13], and climate variability, particularly rainfall anomalies and El Niño cycles, on rice yields [14,15]. Additionally, Moya et al. [16] provided an important long-term perspective based on five decades of household-level surveys of rice farmers, documenting structural changes such as land fragmentation, labour shifts, and varying adoption of modern technologies. While this literature provides important insights into the challenges facing rice production, there remains a lack of contemporary, integrated analysis that combines recent nationwide production data with local contextual understanding. Indeed, few studies have examined how trends vary across regions or how recent government policy interventions have influenced production outcomes.

This study addresses this knowledge gap by analysing regional rice production trends in the Philippines from 2013 to 2023, complemented with qualitative data to uncover the key drivers behind both stagnation and growth. While national-level data show overall stagnation despite rising domestic consumption, our findings reveal substantial regional variation in production trajectories. Importantly, these results suggest that rice production stagnation is not inevitable; by learning from regions that have achieved growth and addressing the factors contributing to decline elsewhere, targeted policy interventions can help revitalize the sector and improve food security outcomes.

The remainder of this paper is structured as follows. First, we provide an overview of our methodology and the datasets we utilized for our analysis. Second, we quantify the domestic rice production deficit and provide a regional overview of rice production over the period 2013–2023. Our analysis continues by highlighting which regions saw either rapid increases or decreases in their rice production over the last five years. We then further analyse these outlier regions using qualitative secondary data, to understand the determinants of these regions’ rapid changes in rice production. In our Discussion we reflect on the overall trends as well as the learnings from regions that have been able to rapidly increase their rice output in recent years.

## Materials and methods

Our methodology progressed in four consecutive steps. First, we used national data on domestic rice production and consumption to quantify the domestic rice production deficit over the last ten years of available data (2012 to 2022), using publicly available data from the Philippine Statistics Authority [17]. The rice production deficit analysis used milled rice production volumes to take into account that there is about a 35% volume loss when unmilled (called ‘palay’ in the Filipino-Tagalog language) is converted to milled rice by removing outer husks and other layers unsuitable for consumption [18]. The second step of our analysis focused on analysing regional differences in rice production, which are also publicly accessible and reported using unmilled (palay) production volumes by the Philippine Statistics Authority [19]. Our regional comparative analysis focused on the three major island groups, Luzon, Visayas, and Mindanao, and on the 17 regions of the Philippines, following the naming convention consistent with the pre-June 2024 Philippine Standard Geographic Code [20]. Regional comparison of rice production focused on volume of production (metric tons) [19], and its underlying causal determinants: area harvested (ha) [21], and yield-per-harvest (metric ton/ha). Volume of production refers to domestic output while area harvested excludes damaged or unproductive plots [22]. Our analysis included rice production from irrigated and rainfed ecosystems. Yield-per-hectare is derived by dividing production by harvest area [18].

All quantitative datasets were consolidated and analyzed using Microsoft Excel and QGIS (v3.38). Regional production volumes, as well as harvest areas and yield-per-harvest, were presented using both temporal and spatial dimensions. Temporally, we used bar graphs to show rice quantities produced by region for the last ten years of available data (2013–2023). Spatially, we visualized regional differences in rice quantities produced for the last year of available data (2023) using QGIS. The Philippine Geopackage from GADM v4.1 was used to spatially map the 2023 Palay Statistics obtained from the Philippine Statistics Authority [19,21]. The Jenks natural breaks classification was used to cluster the spatial distributions of the 2023 total palay production and 2023 area of palay harvested datasets, while equal interval classification was used to cluster the spatial distribution of the 2023 yield-per-harvest palay statistic. Designing the GIS maps utilized map elements from the maps produced by the United States Department of Agriculture Foreign Agriculture Services [23] and the Philippine Rice Information System [24]. Placing the comprehensive bar graphs together with the GIS maps allowed for a consolidated overview of rice production trends temporally and spatially.

The third step of our analysis used the same longitudinal rice production data by the Philippine Statistics Authority [19,21] to visualize and analyse the relative changes in production volumes, harvest areas, and yield-per-harvest across regions in the last five years (between 2018 and 2023). We evaluated whether there were any regions that were able to rapidly increase their rice production volumes, or vice versa, whether there were any regions that experienced a rapid decrease in rice production. Relative changes were calculated by dividing the difference between the 2018 and 2023 production volumes (or harvest areas, yield-per-harvest) by the 2018 baseline. In the final and fourth step of our analysis, we then used qualitative document analysis to explore the potential causal factors contributing to the rapid growth or decline in regional rice production. Our document analysis included reports and information from both formal literature and secondary information including government reports, news articles, and other relevant documentation found using Google search databases in both English and regional languages such as Tagalog and Ilokano.

## Results

### Quantifying the (milled) rice deficit

**Fig 1:**
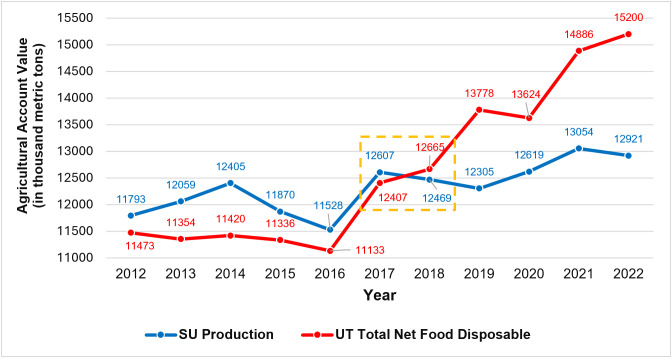
Domestic rice consumption versus (milled) rice production in the Philippines between 2012 and 2022. Supply (SU) Production reflects domestic rice production while Utilization (UT) Total Net Food Disposable reflects rice used in its original form for human consumption. Stagnation in domestic production is highlighted from 2017-2018. Figure was produced by authors using data from the Philippines Statistics Authority [17].

National-level data highlights the widening gap between rice consumption and production in the Philippines (Fig 1). While domestic rice production stagnated from 2017 onwards, domestic rice consumption continued its upward trend that started around 2016. By the year 2022, domestic rice consumption significantly exceeded domestic production. Specifically, by 2022, domestic rice consumption exceeded domestic rice production by 2.3 million metric tons, or by 18%.

### Regional differences in (unmilled) rice production

**Fig 2:**
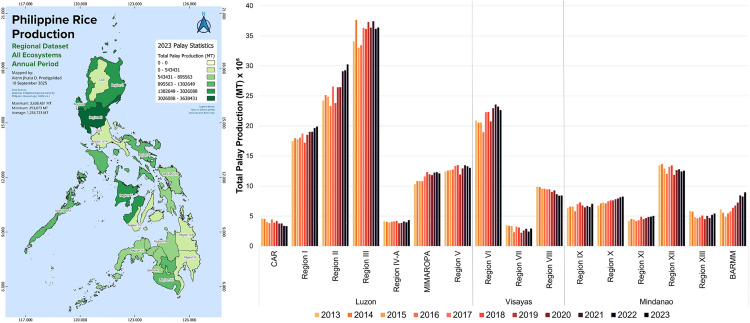
Regional differences in total palay (unmilled rice) production from 2013 to 2023. Spatial distribution of total palay production for 2023 generated using QGIS v3.38. The National Capital Region (NCR) located on Luzon Island is excluded as it is not documented with (unmilled) rice production statistical data. Figure was produced by authors using data from the Philippine Statistics Authority [19]. Larger version of the spatial map can be found in S1 Fig.

Regional-level data highlights noticeable differences in rice production between the three major island groups in the Philippines (Luzon, Visayas, and Mindanao), as well as between specific regions within these major island groups (Fig 2). Total production of palay (unmilled rice) in the Philippines increased from 18.4 million metric tons in 2013 to 20.1 million metric tons in 2023, or a 9% increase. Overall production volumes between the three major island groups have remained relatively stable between 2013 and 2023, with Luzon as the largest rice producer, followed at a distance by Mindanao and Visayas. In 2013, Luzon produced 10.7 million metric tons of rice (58% of the total), followed by 4.3 million metric tons in Mindanao (23% of the total), and 3.4 million metric tons in the Visayas (19% of the total). In 2023, Luzon produced 11.9 million metric tons of rice (59% of the total), followed by 4.7 million metric tons in Mindanao (24% of the total), and 3.4 million metric tons in the Visayas (17% of the total). Within Luzon, Region 1 (Ilocos Region), Region 2 (Cagayan Valley), and Region 3 (Central Luzon) are the leading rice-producing regions. In Visayas, Region 6 (Western Visayas) is the largest producer, while in Mindanao, Region 12 (SOCCSKARGEN) is the largest producer.

**Fig 3:**
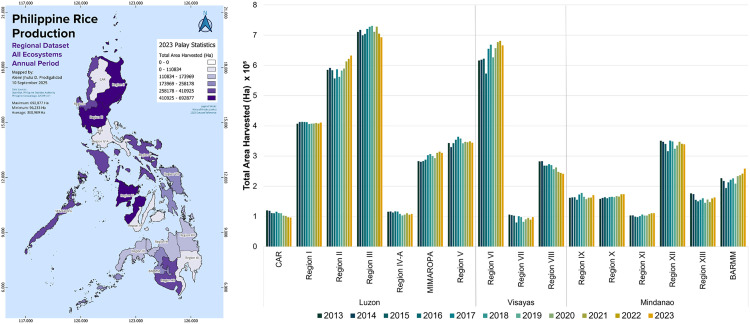
Regional differences in total harvested land for palay (unmilled rice) production from 2013 to 2023. Spatial distribution of total harvested land for palay production for 2023 generated using QGIS v3.38. The National Capital Region (NCR) located on Luzon Island is excluded as it is not documented with (unmilled) rice production statistical data. Figure was produced by authors using data from the Philippine Statistics Authority [21]. Larger version of the spatial map can be found in S2 Fig.

Like the regional rice production volumes (Fig 2), the hectares available for rice farming remained relatively stable over time (Fig 3). Total available farmland for rice farming in the Philippines increased from 4.7 million hectares in 2013 to 4.8 million hectares in 2023, or a 1% increase. In 2013, Luzon had 2.6 million hectares of rice farmland (54% of the total), followed by 1.2 million hectares in Mindanao (25% of the total), and 1.0 million hectares in the Visayas (21% of the total). The total available farmland for rice production across the three major island groups did not noticeably change between 2013 and 2023. The regions within the three major island groups with the largest land available for rice production closely followed the production volumes presented in Fig 2.

**Fig 4:**
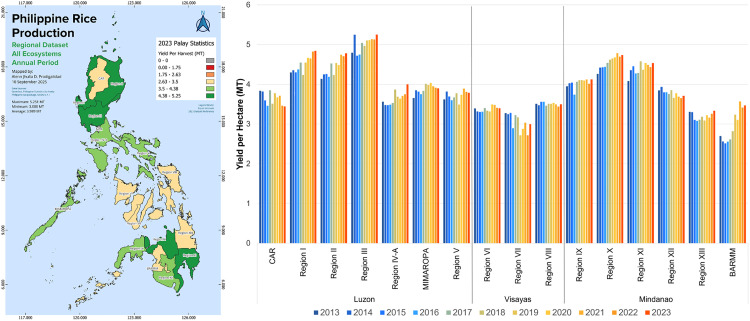
Regional differences in yield for palay (unmilled rice) production from 2013 to 2023. Spatial distribution of yield for palay production for 2023 generated using QGIS v3.38. The National Capital Region (NCR) located on Luzon Island is excluded as it is not documented with (unmilled) rice production statistical data. Figure was produced by authors using data from the Philippine Statistics Authority [19,21]. Larger version of the spatial map can be found in S3 Fig.

The yields-per-harvest (metric tons of rice produced per hectare of farmland) overall showed pronounced geographical differences between the three major island groups and between specific regions within these island groups (Fig 4). Some regions in Mindanao exhibited higher yields comparable to top rice-producing regions in Luzon, while there was also a noticeable clustering of regions with relatively lower yields that included most of the Visayas island group and parts of Northern Mindanao. The average yield across the Philippines increased from 3.9 in 2013 to 4.2 in 2023, or a 7% increase. The island group of Luzon had overall the highest yields, followed by Mindanao and Visayas. Yields per hectare of rice farmland increased between 2013 and 2023 in Luzon and Mindanao, while they remained stagnant in Visayas. In 2013, the rice yield was 4.2 metric tons per hectare of farmland in Luzon, 3.6 in Mindanao, and 3.4 in Visayas. In 2023, the rice yield was 4.6 in Luzon, 3.9 in Mindanao, and 3.4 in Visayas. Differences in yields between regions in 2023 ranged from 3.0 in Region 7 (Central Visayas) to 5.3 in Region 3 (Central Luzon).

Disaggregated production, area harvested, and yield data by ecosystem, either irrigated or rainfed, have been provided in the S9–S14 Figs. These graphs show that irrigated rice fields overall provided the largest share of rice production, amounting to 15.3 million metric tons in 2023, or 76% of the total production (S9 Fig, S10 Fig). Rice production from irrigated rice fields also had higher yields across all regions compared to production from rainfed ecosystems (S13 Fig, S14 Fig). Notably, some regions had atypical rainfed ecosystem productivity. Western Visayas (Region VI) had by far the highest rice production volume and farmlands from rainfed ecosystems (S10 Fig, S12 Fig).

### Relative changes in (unmilled) rice production between 2018–2023

**Fig 5:**
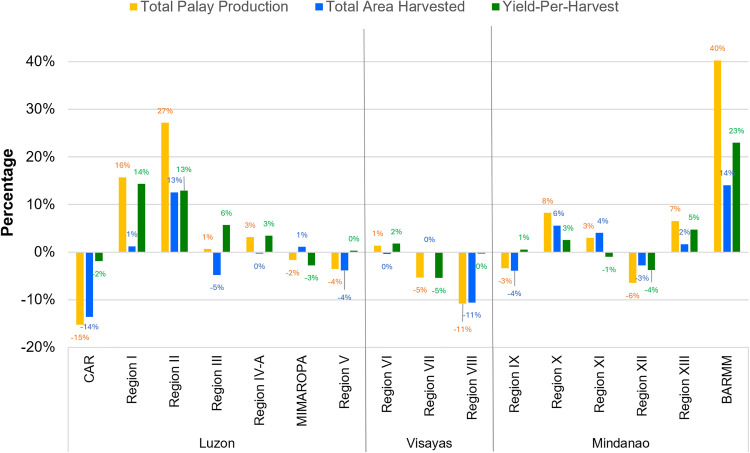
Relative changes in regional total palay production, area harvested, and yield-per-harvest in all ecosystems. Relative changes documented between 2018 and 2023. The National Capital Region (NCR) located on Luzon Island is excluded as it is not documented with (unmilled) rice production statistical data. Figure was produced by authors using data from the Philippine Statistics Authority [19,21].

In terms of relative changes in palay (unmilled rice) production over the last five years of available data (2018 to 2023), we find that three regions experienced a rapid increase in their rice output, while two regions experienced a noticeable decrease (Fig 5). The Bangsamoro Autonomous Region in Muslim Mindanao (BARMM) saw the strongest relative increase in rice production volumes, by 40% over the last five years. Region 2 (Cagayan Valley) and Region 1 (Ilocos Region) in Luzon also saw strong relative increases in rice production, with rice production volumes increasing respectively 27% and 16% between 2018 and 2023. The relative increase in production volumes in BARMM and Region 2 (Cagayan Valley) was attributed to both increasing farmland and yields, while in Region 1 (Ilocos Region) the relative increase was attributed mainly to increased yields. The Cordillera Administrative Region (CAR) and Region 8 (Eastern Visayas) saw the strongest relative decrease in rice production, with volumes decreasing with respectively 15% and 11% between 2018 and 2023 (Fig 5). In both CAR and Region 8 (Eastern Visayas), the relative decrease was mainly attributed to a loss of available farmland for rice production.

The absolute changes of total palay production and total area harvested between 2018–2023 per region can be viewed in S4 Fig and S5 Fig respectively. Meanwhile, the absolute changes of yields-per-harvest between 2018 to 2023 per region can be viewed in S6 Fig.

### Causal factors contributing to rice production growth and decline

The Philippine rice production stagnation from 2018 onwards (Fig 1) masked significant regional differences seen in Fig 2 and Fig 5. BARMM (Mindanao) and Regions I and II (Luzon) exhibited significant increases in their rice production volumes, while CAR (Luzon) and Region VIII (Visayas) revealed significant decreases in their total rice production. We describe potential causes for the rapid changes in these specific regions because they could provide insights into how further rice production growth in the Philippines can be stimulated and/or how further loss of rice production can be prevented or reversed.

Rice production in BARMM (Mindanao) increased from 0.64 million metric tons in 2018 to 0.90 million metric tons in 2023, or a 40% increase (Fig 5). The rapid expansion of rice production occurred in irrigated and rainfed ecosystems. BARMM’s irrigated rice production increased from 0.16 million metric tons in 2018 to 0.29 million metric tons in 2023 (or an 83% increase; S7 Fig), while its rainfed rice production increased from 0.48 million metric tons in 2018 to 0.61 million metric tons in 2023 (or a 26% increase; S8 Fig). The increase in rice production in both irrigated and rainfed ecosystems in BARMM (S9 Fig; S10 Fig) was caused by both expanding farmlands (S11 Fig; S12 Fig) and increasing yields (S13 Fig; S14 Fig). BARMM’s growing rice production could potentially be attributed to the harmonized interventions of the Ministry of Agriculture, Fisheries, and Agrarian Reform (MAFAR), a regional executive department dedicated to overseeing BARMM’s agriculture that was established in 2019, which includes partnerships with the Food and Agriculture Organization of the United Nations [25,26]. The region also received high-quality inbred rice seeds in 2019, through the Rice Competitiveness Enhancement Fund (RCEF), which may have contributed to increasing rice yields [27]. In 2023, the region received further assistance in the form of rice processing systems under the RCEF’s Mechanization Program [28], the outcomes of which are not yet visible in the rice production data.

Rice production in the Ilocos Region (Region 1) increased from 1.72 million metric tons in 2018 to 1.99 million metric tons in 2023, or a 16% increase (**Fig 5**). The increase in rice production likewise occurred both in irrigated and rainfed ecosystems, with irrigated rice production increasing from 1.26 million metric tons in 2018 to 1.42 million metric tons in 2023 (or an 13% increase; S7 Fig) and rainfed rice production increasing from 0.46 million metric tons in 2018 to 0.57 million metric tons in 2023 (or a 22% increase; S8 Fig). The increase in rice production in both irrigated and rainfed ecosystems in the Ilocos Region (S9 Fig; S10 Fig) was caused by increasing yields (S13 Fig; S14 Fig) because available farmland remained constant (S11 Fig; S12 Fig). The increasing yields could potentially be attributed to the provision of hybrid/inbred rice seeds through the RCEF Seed Program [29]. In 2023, the region received further assistance in the form of rice processing systems supported by the RCEF Mechanization Program [30], the outcomes of which are not yet visible in the rice production data.

Rice production in Cagayan Valley (Region 2) increased from 2.38 million metric tons in 2018 to 3.03 million metric tons in 2023, or a 27% increase (Fig 5). The rapid expansion of rice production was most evident in irrigated ecosystems compared to rainfed ecosystems. Its irrigated rice production increased from 2.16 million metric tons in 2018 to 2.75 million metric tons in 2023 (or a 27% increase; S7 Fig), while its rainfed rice production increased from 0.22 million metric tons in 2018 to 0.27 million metric tons in 2023 (or a 25% increase; S8 Fig). The increase in rice production in both irrigated and rainfed ecosystems in the Cagayan Valley (S9 Fig; S10 Fig) was caused by both expanding farmlands (S11 Fig; S12 Fig) and increasing yields (S13 Fig; S14 Fig). Being home to the Cagayan River, the longest river in the Philippines, Region II’s rice production heavily relies on irrigated lands [31,32]. The expansion of rice farmland in the region could potentially be attributed to the expansion of irrigation areas across the region [33,34]. The increasing yields could potentially be attributed to the introduction of improved clusters for hybrid rice implemented through the Department of Agriculture [34–36].

Rice production in the Cordillera Administrative Region (CAR) decreased from 0.39 million metric tons in 2018 to 0.33 million metric tons in 2023, or a 15% decrease (Fig 5). The decrease in rice production occurred primarily in irrigated ecosystems, with its irrigated rice production decreasing from 0.35 million metric tons in 2018 to 0.29 million metric tons in 2023 (or an 18% decrease; S7 Fig). Meanwhile, rainfed rice production increased from 0.04 million metric tons in 2018 to 0.05 million metric tons in 2023 (or an 8% increase; S8 Fig). The decrease in rice production in irrigated ecosystems in CAR (S9 Fig) was caused mainly by a decrease in farmland available for rice production (S11 Fig) alongside stagnant rice yields in that same ecosystem (S13 Fig). In rainfed ecosystems, a similar decline in farmland available for rice production was observed (S12 Fig) without a subsequent decline in rice production (S10 Fig) because of improvements in rice yields (S14 Fig). CAR is the only land-locked region in the Philippines, being mountainous and home to rice terraces, some being world heritage sites, but most are non-functional for national commercial use [37]. The loss of rice farmland in the region could potentially be attributed to the lack of investment in irrigation infrastructure upgrading and government investment in the region [38], as well as to the impacts from unfavourable weather conditions (e.g., El Niño, La Niña, Habagat) and recent typhoons (e.g., Typhoons Egay and Falcon) [28].

Rice production in Eastern Visayas (Region VIII) decreased from 0.95 million metric tons in 2018 to 0.84 million metric tons in 2023, or a 11% decrease (Fig 5). The decrease in rice production occurred in both rainfed and irrigated ecosystems, with irrigated rice production decreasing from 0.53 million metric tons in 2018 to 0.50 million metric tons in 2023 (or a 7% decrease; S7 Fig) and rainfed rice production decreasing from 0.41 million metric tons in 2018 to 0.35 million metric tons in 2023 (or a 16% decrease; S8 Fig). The decrease in rice production in both irrigated and rainfed ecosystems in Eastern Visayas (S9 Fig; S10 Fig) was caused mainly by a decrease in farmland available for rice production (S11 Fig; S12 Fig) alongside stagnant rice yields (S13 Fig; S14 Fig). The loss of rice farmland in the region could potentially be attributed to the frequent occurrence of natural calamities, especially typhoons and drought conditions [39,40]. Moreover, there is competition for farmland in the region between rice and coconut, which may have higher profitability in terms of its export markets [41,42].

## Discussion

Our consolidated regional analysis of rice production in the Philippines provides new insights to better understand the drivers of the increasing rice deficit in the country. Our analysis provided three key findings. First, we found that most of the regions in the Philippines indeed experienced a stagnation in their rice production volumes (Fig 2 and Fig 5), because of stagnant farmland areas (Fig 3) and stagnant crop yields (Fig 4). Compared to prior studies on the causes of rice stagnation, we did not find strong evidence that urbanization-driven farmland loss was a major driver of the stagnation between 2013 and 2023, which was proposed in an earlier study by Bravo [11]. Second, we found that despite widespread stagnation in rice production, some regions were able to grow their production output rapidly over short periods of time and driven by dedicated and harmonized government programs (BARMM), expansion of irrigation infrastructure (Cagayan Valley) and switching to more productive seed varieties (BARMM, Cagayan Valley, and Ilocos Region). Third, we found that rapidly declining rice production in other regions could be attributed to climate change and natural calamities (CAR and Eastern Visayas), lack of infrastructure investments (CAR), and competition with other agricultural products such as coconuts (Eastern Visayas). Our findings thus provide some support for prior hypotheses about the role of natural disasters in declining rice production [12] but we did not find an indication that soil erosion was a major contributor [13]. Our finding that shifts to more financially attractive land uses, such as cultivating coconuts instead of rice, can negatively impact food security aligns with evidence from other regions across the Global South [43–45].

Our findings complement prior work done by Yuan et al. [5] that identified large yields gaps in the Philippines based on theoretically possible yields and actual yields achieved by farmers. We found a noticeable clustering of lower-yield rice-producing regions, covering most of the Visayas island group and parts of Mindanao (Fig 4), which requires further research to be explained in terms of their deviation from other higher-yield regions. However, we found that there is potential for rapid gains in rice yields, as have been observed over the last five years in Cagayan Valley and BARMM (Fig 5). Whether these yield improvements will be significant and widespread remains an open question. Moreover, it may be challenging for the Philippines to close the rice deficit gap (Fig 1) without expansions in land available for rice farming. As noted by Yuan et al. [5], there is likely only limited potential for cropland expansion in Southeast Asia, including the Philippines. Optimistically, our findings indicated that rapid cropland expansion was possible and can be driven by the extension and improvement of irrigation systems where sufficient water sources are available, such as for the Cagayan River in Region II, Luzon. Additionally, there may be significant potential left for cropland expansion in Mindanao, which is almost equal in size to Luzon, but has only half the available farmland for rice production. Our evidence from BARMM shows that dedicated government programs can lead to increased efficiency and profitability of rice farming and rapidly increase farmland development (Fig 5).

Our findings provide empirical evidence for existing impacts from climate change on food security in the context of the Global South [46–49]. Specifically, we found that climate-related natural hazards, such as increasingly severe droughts and typhoons, contributed to farmland loss, for example in the Cordillera Administrative Region and Eastern Visayas (Fig 5). While every rice-producing region in the Philippines is to some extent vulnerable to the adverse impacts of El Niño, La Niña, and typhoons, each region mitigates low rice productivity differently depending on its geographical, technological, and financial resources [50–52]. While existing support mechanisms for rice farmers exist under the Rice Competitiveness Enhancement Fund (RCEF), it is unclear whether the mechanisms they provide are sufficiently focused on developing climate-resilient rice production methods which would help mitigate increasingly severe impacts from climate change on rice farmers across the country, and especially in highly vulnerable regions in the Eastern Visayas and Northern Luzon.

### Implications for policy

The Rice Tariffication Law (RTL) was enacted to address low rice productivity, and the Rice Competitiveness Enhancement Fund (RCEF) was established as a corollary to the RTL to incentivize farmers to increase their productivity. The nationwide fund comprises four government-led interventions: Mechanization Program to improve rice production-harvesting technology; Seed Program to introduce better varieties of rice that provide better yields; Credit Program that allows farmers to avail loans, and; Extension Services Program that provides rice training and education [53]. The RCEF is implemented in Regions I, II, VIII, CAR, and BARMM mostly through the Rice Farmers Financial Assistance (RFFA), Seed, Mechanization, and Extension Services as documented by news outlets and government websites. However, the RCEF remains insufficient to address low rice productivity as observed in Fig 5 through Region VIII and CAR. The additional interventions of regional offices in Region II (Department of Agriculture Region 2) and in BARMM (MAFAR) contributed to these regions’ growing rice productivity.

While the Rice Competitiveness Enhancement Fund (RCEF), established under Republic Act 11203, was extended through RA 12078 until the year 2031 [54], several evaluations have raised concerns about its implementation. The Commission on Audit flagged inefficiencies in fund disbursement and procurement by the Philippine Center for Postharvest Development and Mechanization (PhilMech), including delays and underutilization of distributed machinery [55]. While some program components, such as seed distribution, have shown improvements in delivery timeliness [56], broader assessments suggest that RCEF has not consistently translated into improved farmer incomes or productivity. Cruz highlights structural challenges in Philippine agricultural sector, including landlessness, weak market linkages, and inadequate public investment, that limit the effectiveness of programs like RCEF [57]. These findings suggest a gap between RCEF’s policy goals and its actual outcomes on the ground. Acknowledging these challenges is essential for a balanced analysis of RCEF’s role in Philippine agricultural development.

Our research highlights the need for a more efficient and holistic approach to solve the rice stagnation problem in the Philippines, one that integrates seed and mechanization interventions with infrastructure investments, financial incentives (e.g., to reduce expenditure on oil and fertilizer), and capacity-building programs. Moreover, the limited duration and funding allocation of the Competitiveness Enhancement Fund (RCEF) could mean that sustainable productivity gains will be harder to achieve [10,58]. Solely relying on the national RCEF does not secure continuous development if it is not supplemented with regional interventions such as those enacted in BARMM and the Cagayan Valley. There are also questions about the RCEF’s long-term sustainability, particularly with the possible over-mechanization of rice, hybrid rice over-consumption, and cash assistance limitations. Hence, future research should be conducted with regional stakeholders and farmers to better understand regional support preferences related to increasing rice productivity, preferably using control groups with farming areas that did not receive RCEF support.

## Supporting information

S1 Fig 2023 Total Palay Production Volume using all ecosystems in an annual period. Figure was produced using data from the Philippine Statistics Authority [19].
(TIF)

S2 Fig 2023 Total Area of Palay Harvested using all ecosystems in an annual period. Figure was produced using data from the Philippine Statistics Authority [21].
(TIF)

S3 Fig 2023 Palay Yield-Per-Harvest using all ecosystems in an annual period. Figure was produced using data from the Philippine Statistics Authority [19,21].
(TIF)

S4 Fig Absolute changes of regional rice production volume in all ecosystems between 2018 and 2023. Figure was produced using data from the Philippine Statistics Authority [19].
(TIF)

S5 Fig Absolute changes of regional area of palay harvest in all ecosystems between 2018 and 2023. Figure was produced using data from the Philippine Statistics Authority [21].
(TIF)

S6 Fig Absolute changes of regional yield-per-harvest in all ecosystems between 2018 and 2023. Figure was produced using data from the Philippine Statistics Authority [19,21].
(TIF)

S7 Fig Relative changes in regional total palay production, area harvested, and yield-per-harvest in rainfed ecosystems. Relative changes documented between 2018 and 2023. Figure was produced using data from the Philippine Statistics Authority [19,21].
(TIF)

S8 Fig Relative changes in regional total palay production, area harvested, and yield-per-harvest in irrigated ecosystems. Relative changes documented between 2018 and 2023. Figure was produced using data from the Philippine Statistics Authority [19,21].
(TIF)

S9 Fig Total rice production volume in irrigated ecosystems by region. Figure was produced using data from the Philippine Statistics Authority [19].
(TIF)

S10 Fig Total rice production volume in rainfed ecosystems by region. Figure was produced using data from the Philippine Statistics Authority [19].
(TIF)

S11 Fig Total area of palay harvest in irrigated ecosystems by region. Figure was produced using data from the Philippine Statistics Authority [21].
(TIF)

S12 Fig Total area of palay harvest in rainfed ecosystems by region. Figure was produced using data from the Philippine Statistics Authority [21].
(TIF)

S13 Fig Palay Yield-Per-Harvest in irrigated ecosystems by region. Figure was produced using data from the Philippine Statistics Authority [19,21].
(TIF)

S14 Fig Palay Yield-Per-Harvest in rainfed ecosystems by region. Figure was produced using data from the Philippine Statistics Authority [19,21].
(TIF)

S1 File Dataset. (XLSX)

S2 File Production 2E4EVCP0. (CSV)

S3 SU UT 2B5FSUA0. (CSV)
