# Supplementary figures and images for: Understanding rice production stagnation in the Philippines: Regional evidence and development implications
Source: PLoS One. 2025 Oct 24;20(10):e0335344. doi: 10.1371/journal.pone.0335344 (PMC12551908; doi:10.1371/journal.pone.0335344)

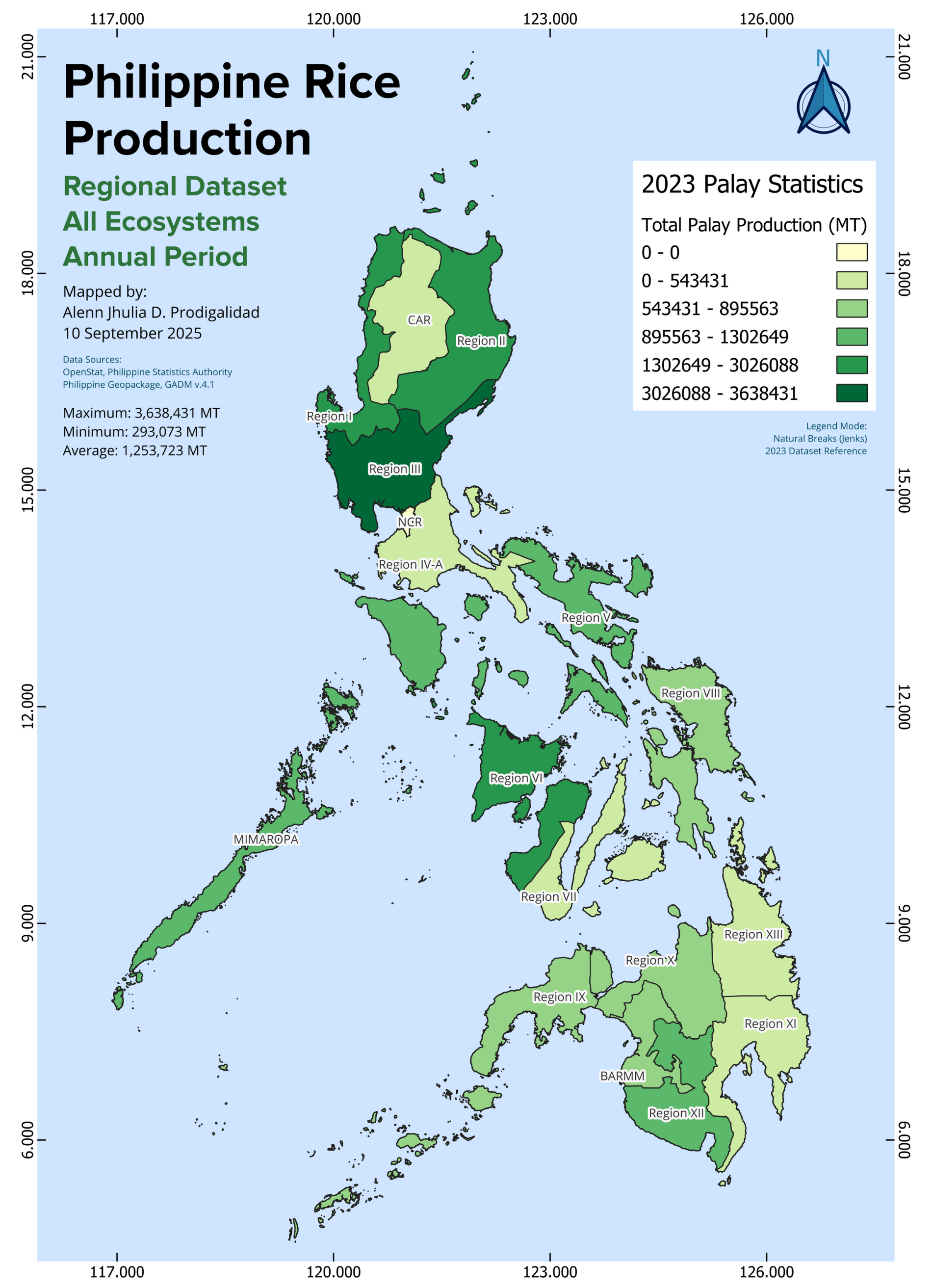

Supplement: S1 Fig — Figure was produced using data from the Philippine Statistics Authority [19]. (TIF) [file pone.0335344.s001.tif]

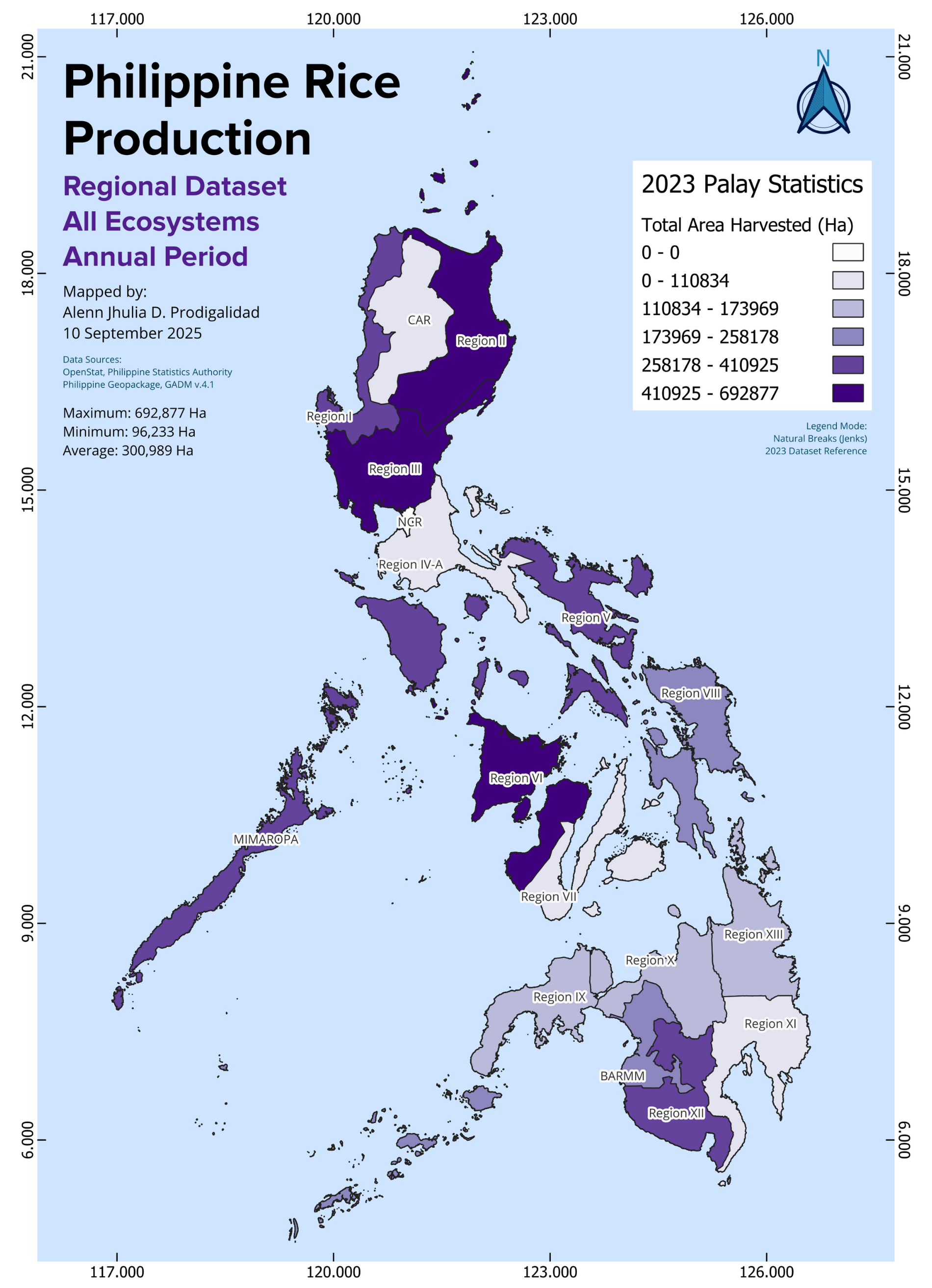

Supplement: S2 Fig — Figure was produced using data from the Philippine Statistics Authority [21]. (TIF) [file pone.0335344.s002.tif]

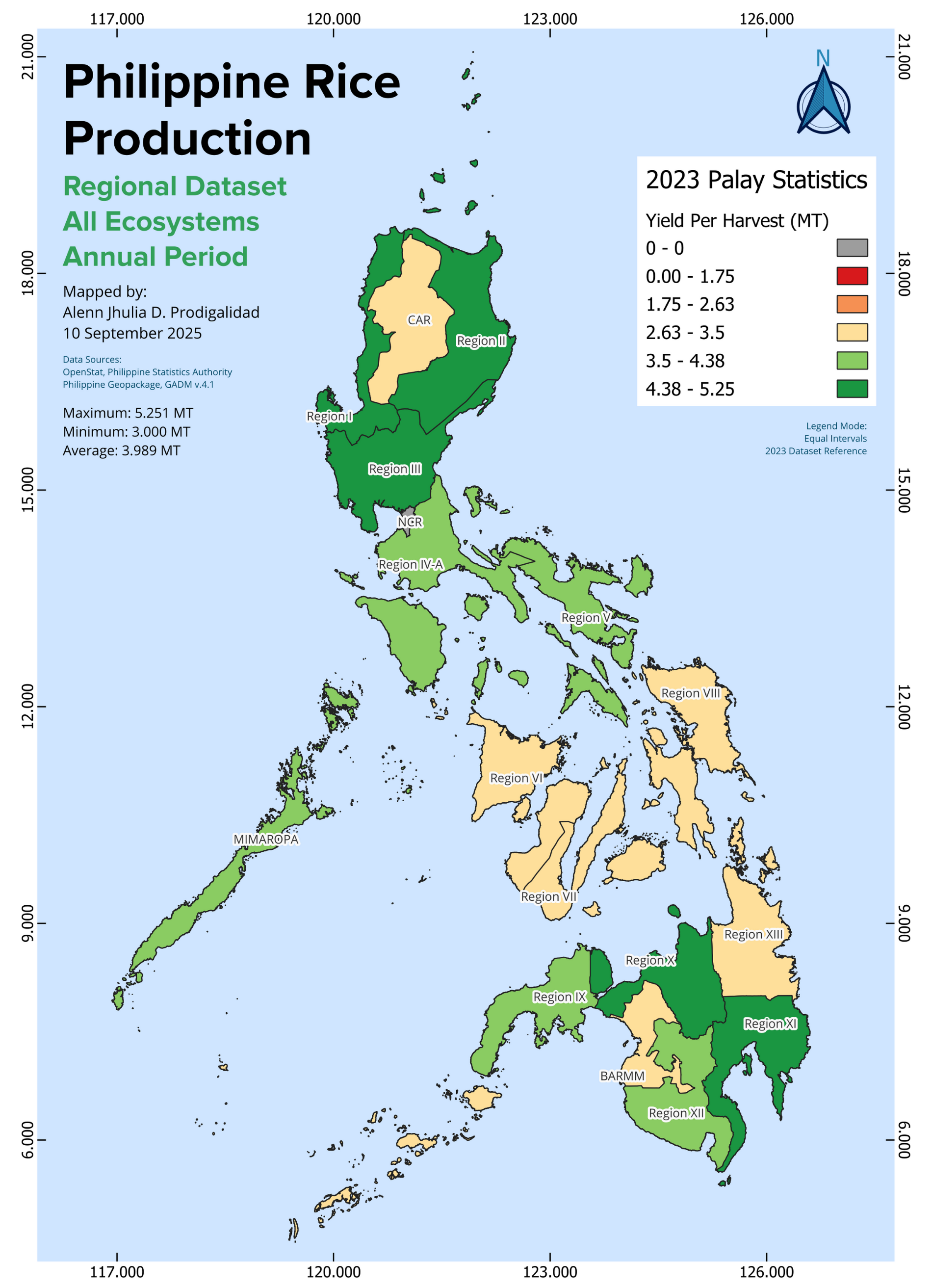

Supplement: S3 Fig — Figure was produced using data from the Philippine Statistics Authority [19,21]. (TIF) [file pone.0335344.s003.tif]

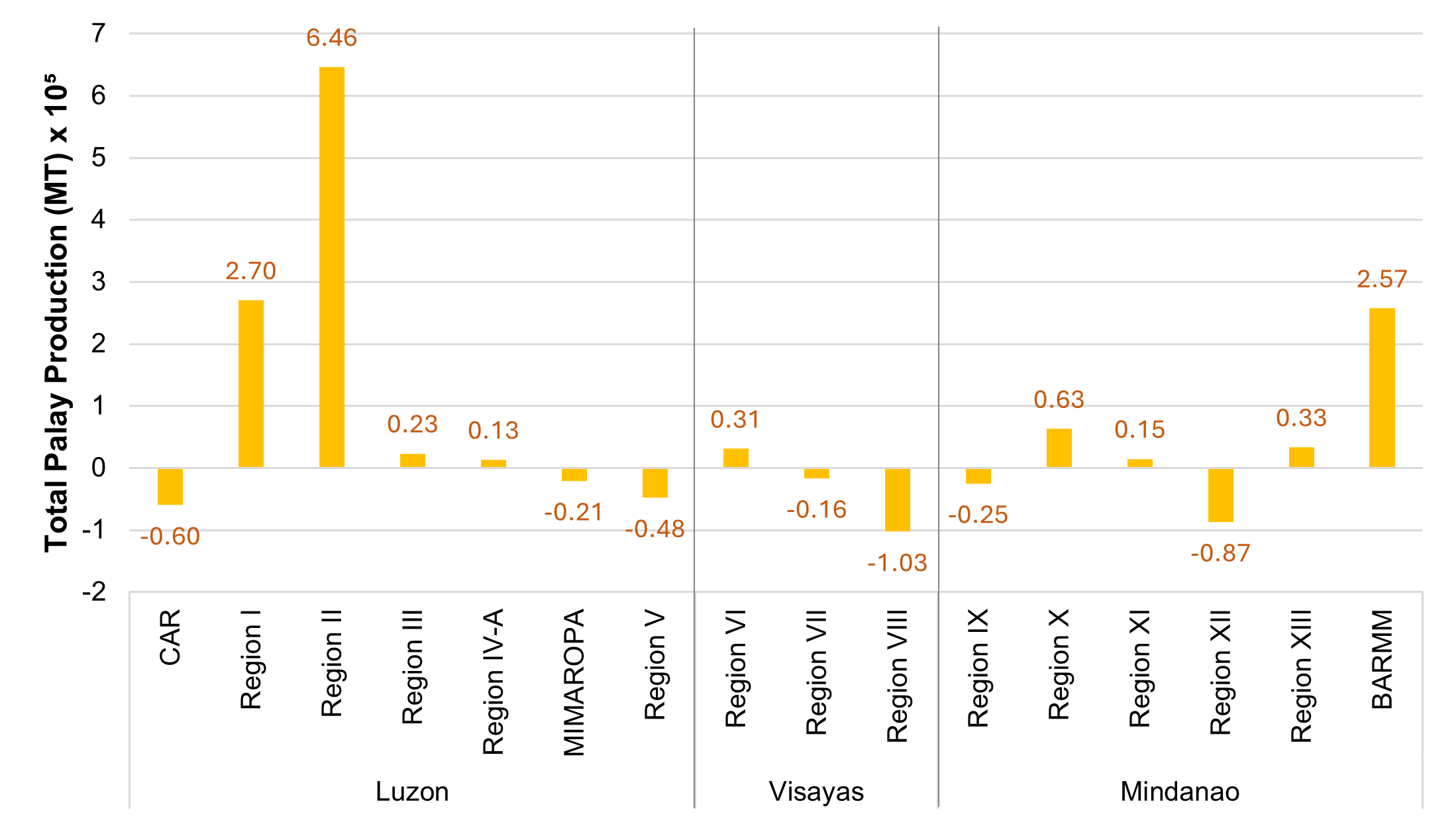

Supplement: S4 Fig — Figure was produced using data from the Philippine Statistics Authority [19]. (TIF) [file pone.0335344.s004.tif]

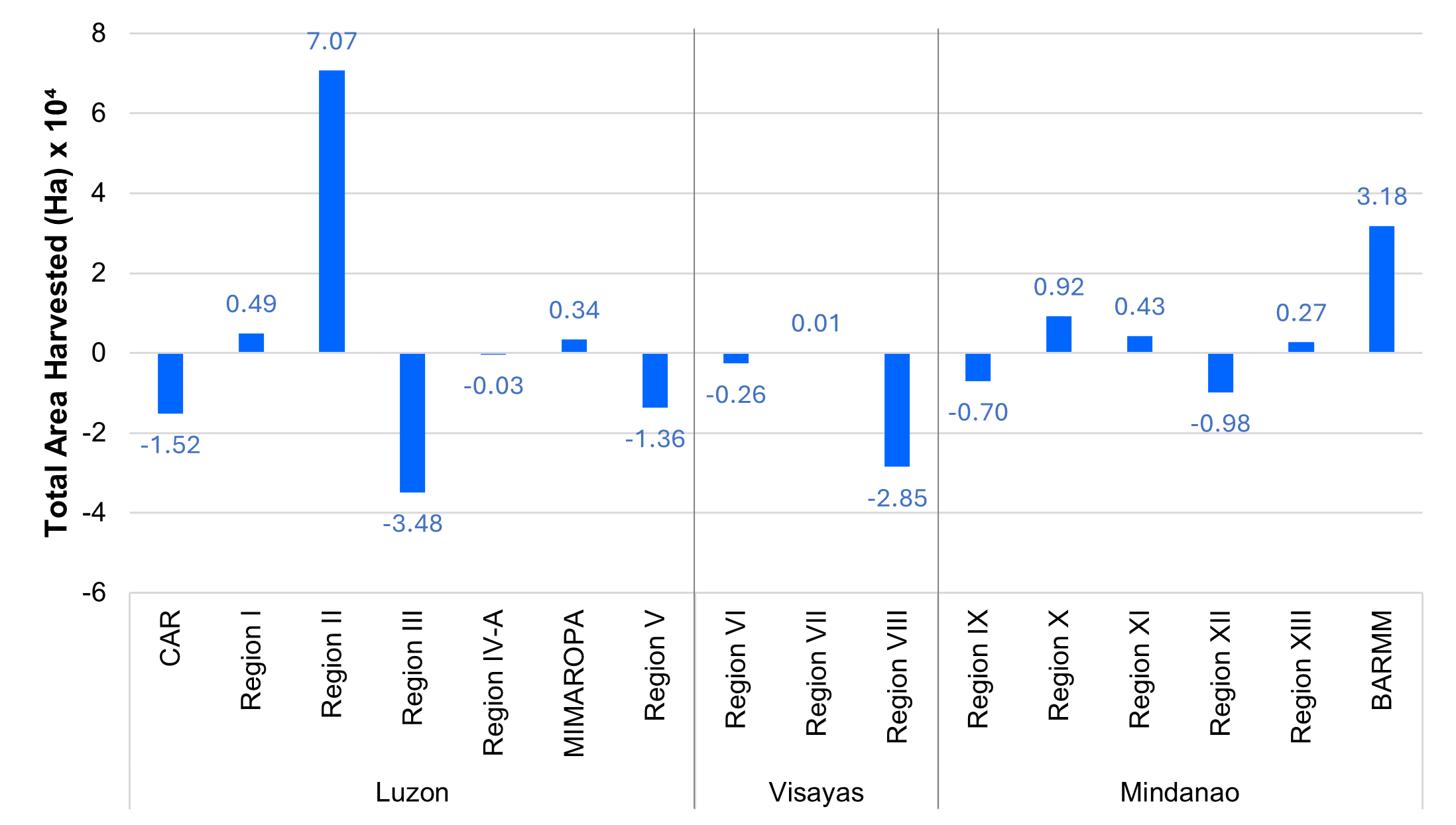

Supplement: S5 Fig — Figure was produced using data from the Philippine Statistics Authority [21]. (TIF) [file pone.0335344.s005.tif]

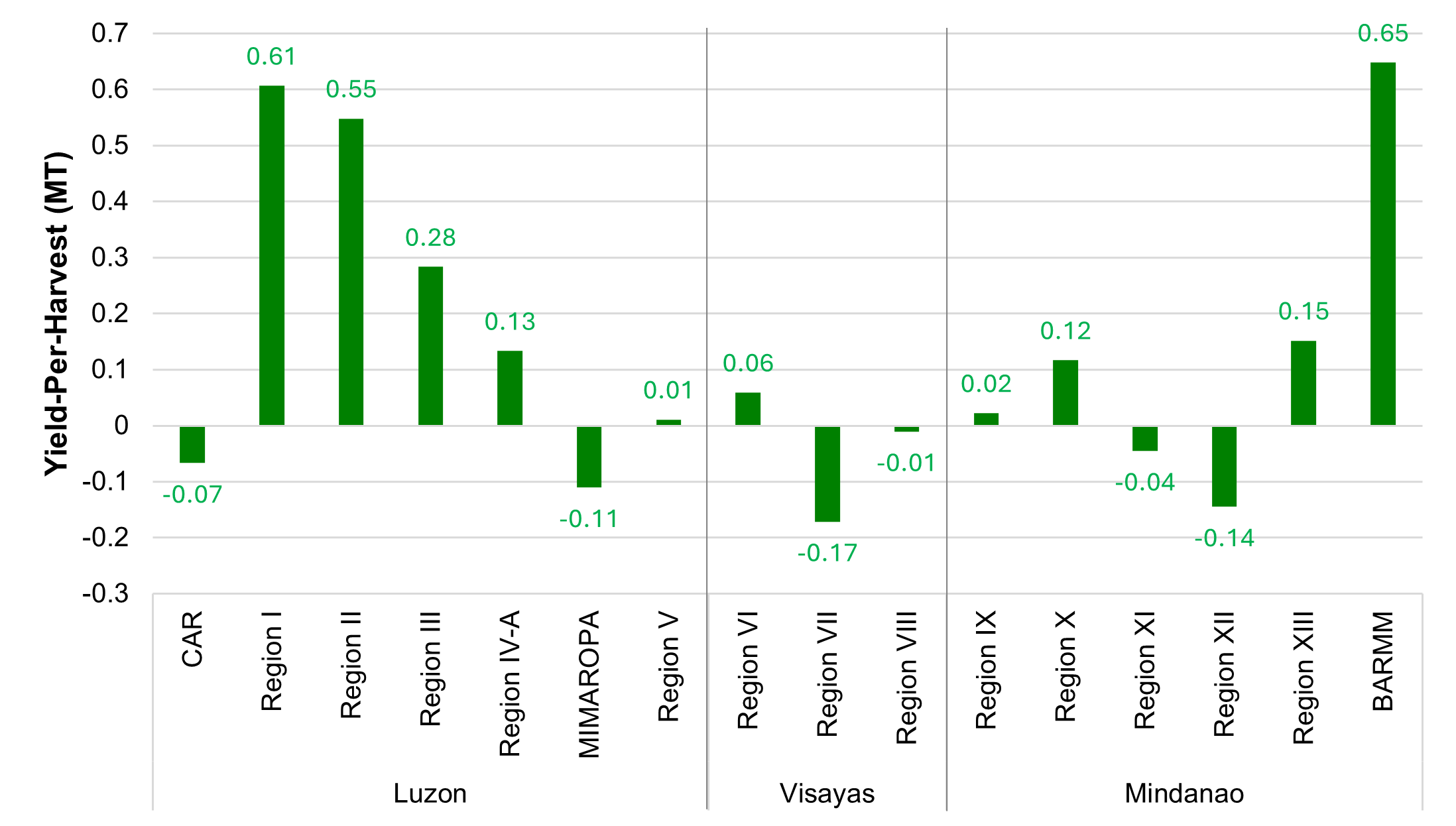

Supplement: S6 Fig — Figure was produced using data from the Philippine Statistics Authority [19,21]. (TIF) [file pone.0335344.s006.tif]

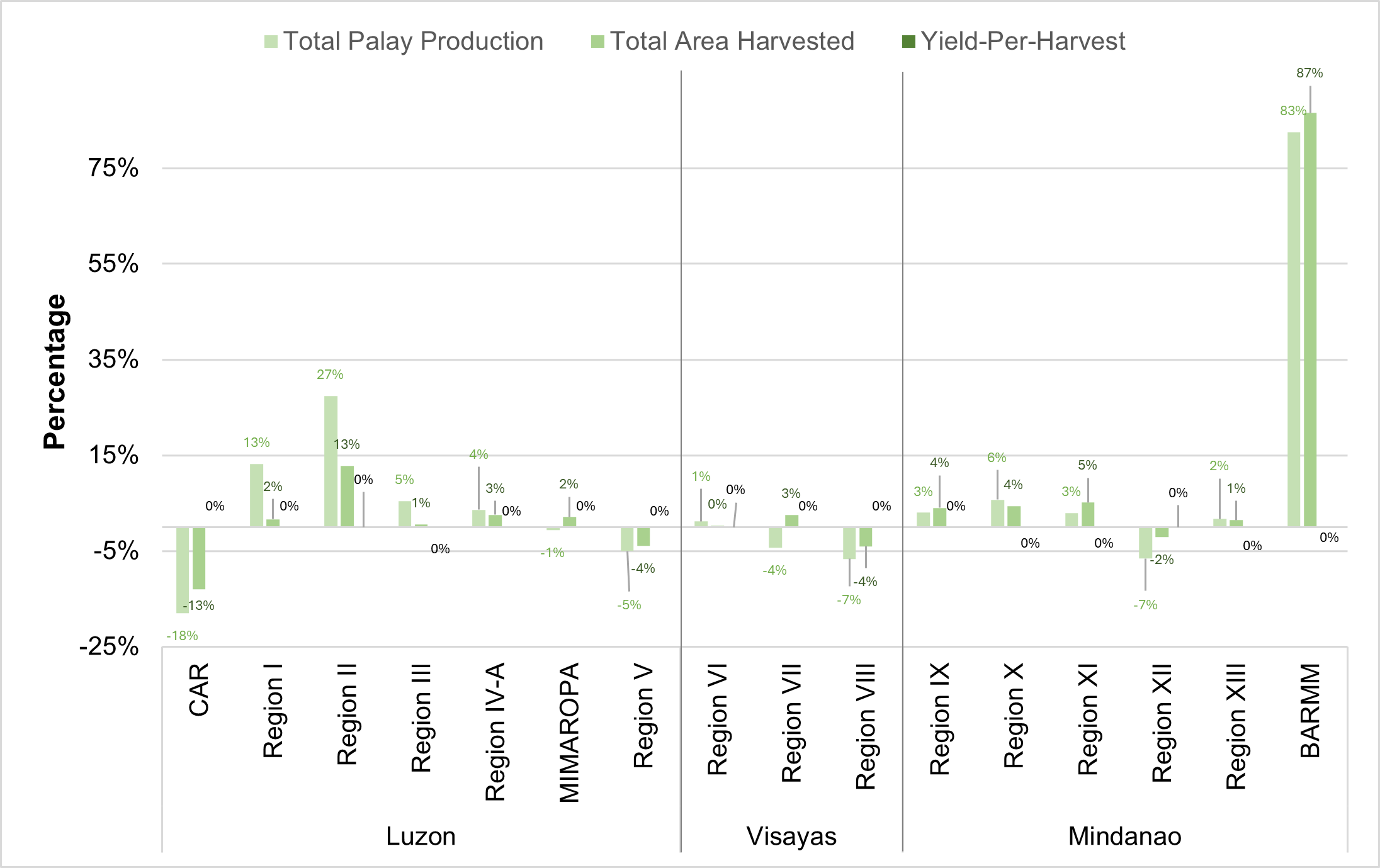

Supplement: S7 Fig — Relative changes documented between 2018 and 2023. Figure was produced using data from the Philippine Statistics Authority [19,21]. (TIF) [file pone.0335344.s007.tif]

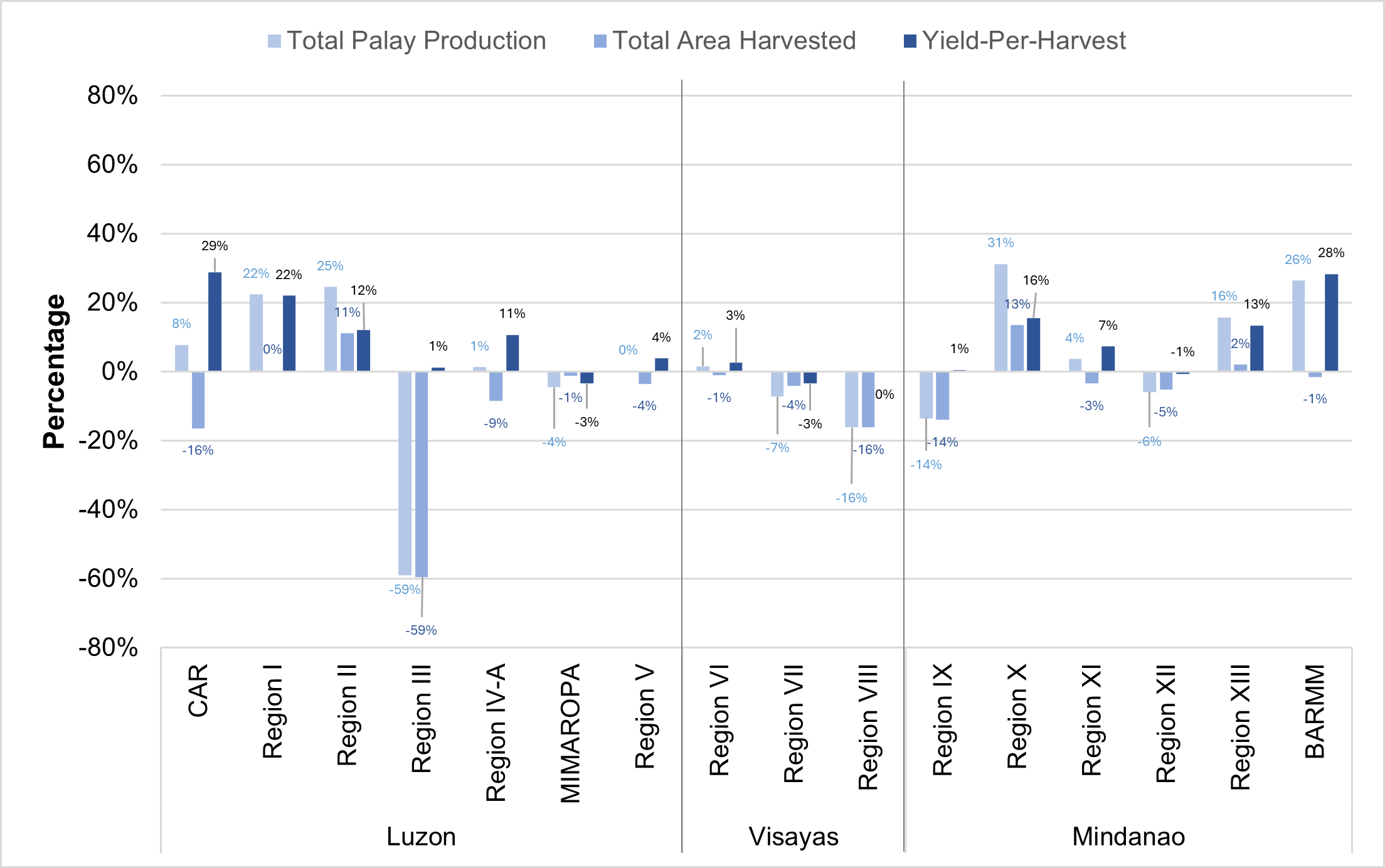

Supplement: S8 Fig — Relative changes documented between 2018 and 2023. Figure was produced using data from the Philippine Statistics Authority [19,21]. (TIF) [file pone.0335344.s008.tif]

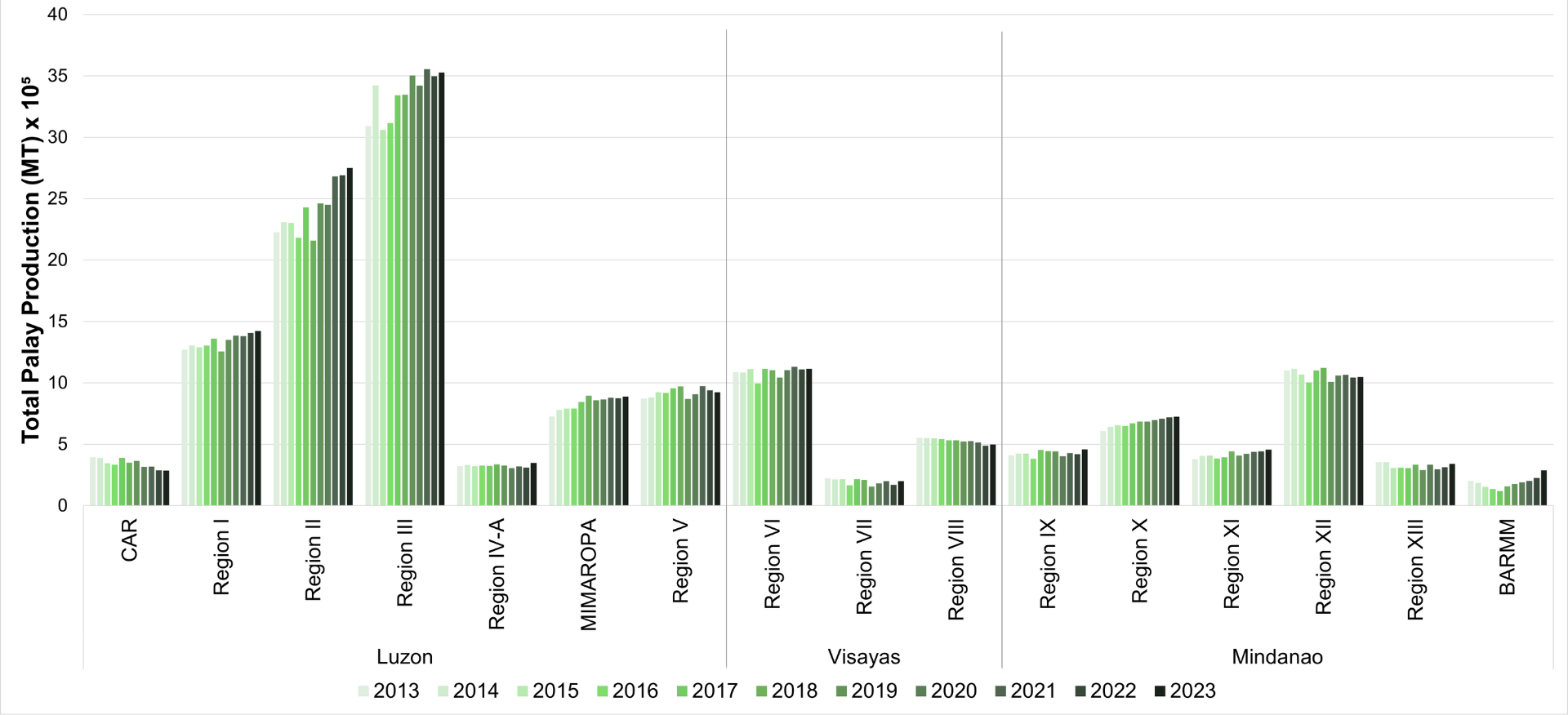

Supplement: S9 Fig — Figure was produced using data from the Philippine Statistics Authority [19]. (TIF) [file pone.0335344.s009.tif]

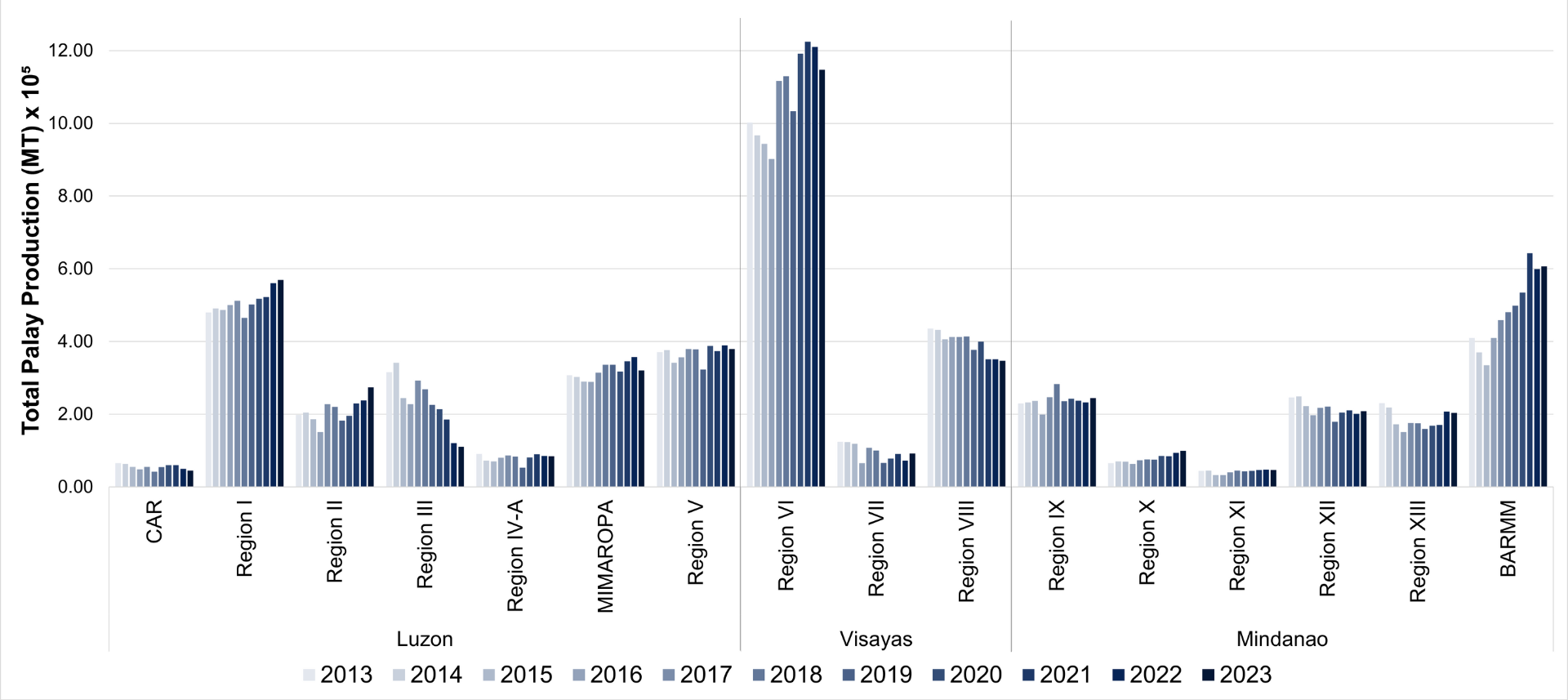

Supplement: S10 Fig — Figure was produced using data from the Philippine Statistics Authority [19]. (TIF) [file pone.0335344.s010.tif]

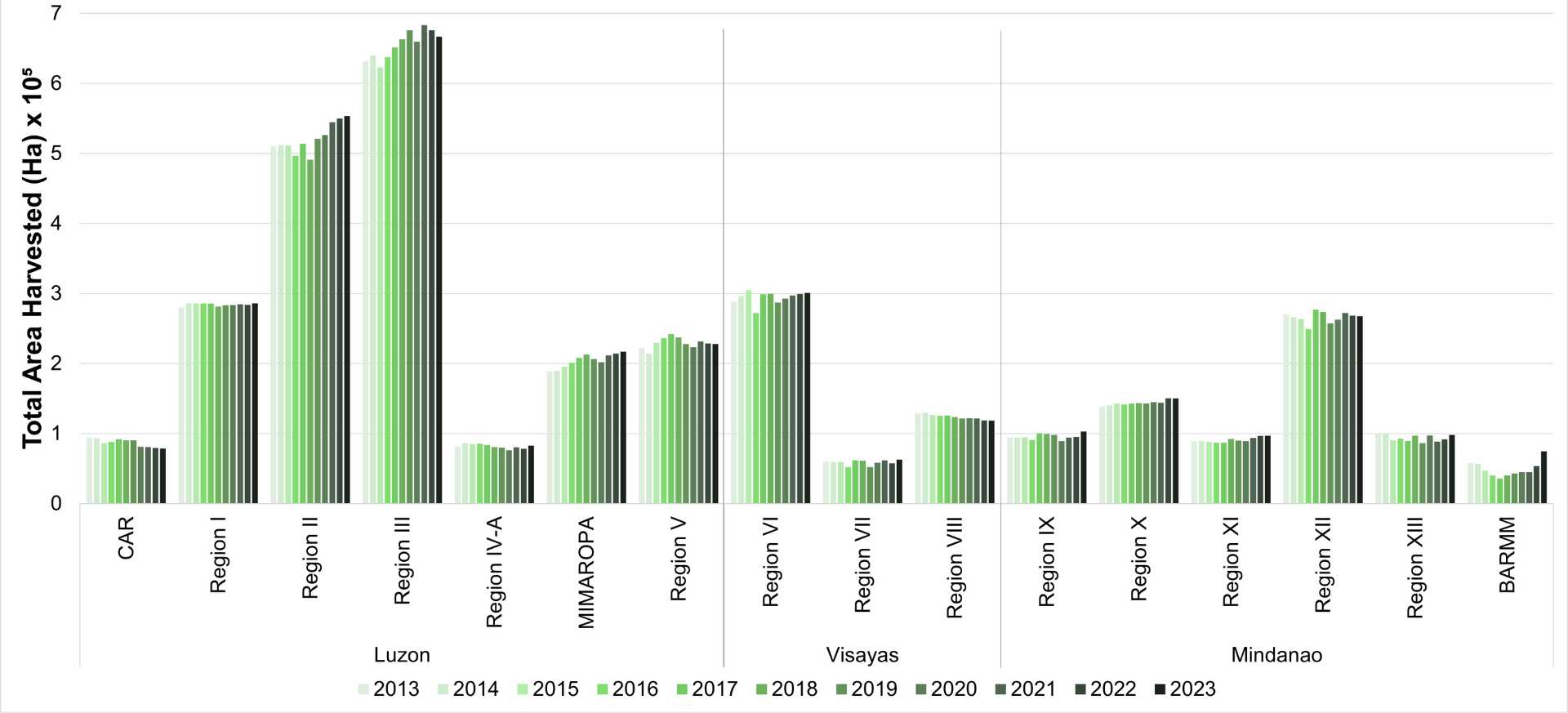

Supplement: S11 Fig — Figure was produced using data from the Philippine Statistics Authority [21]. (TIF) [file pone.0335344.s011.tif]

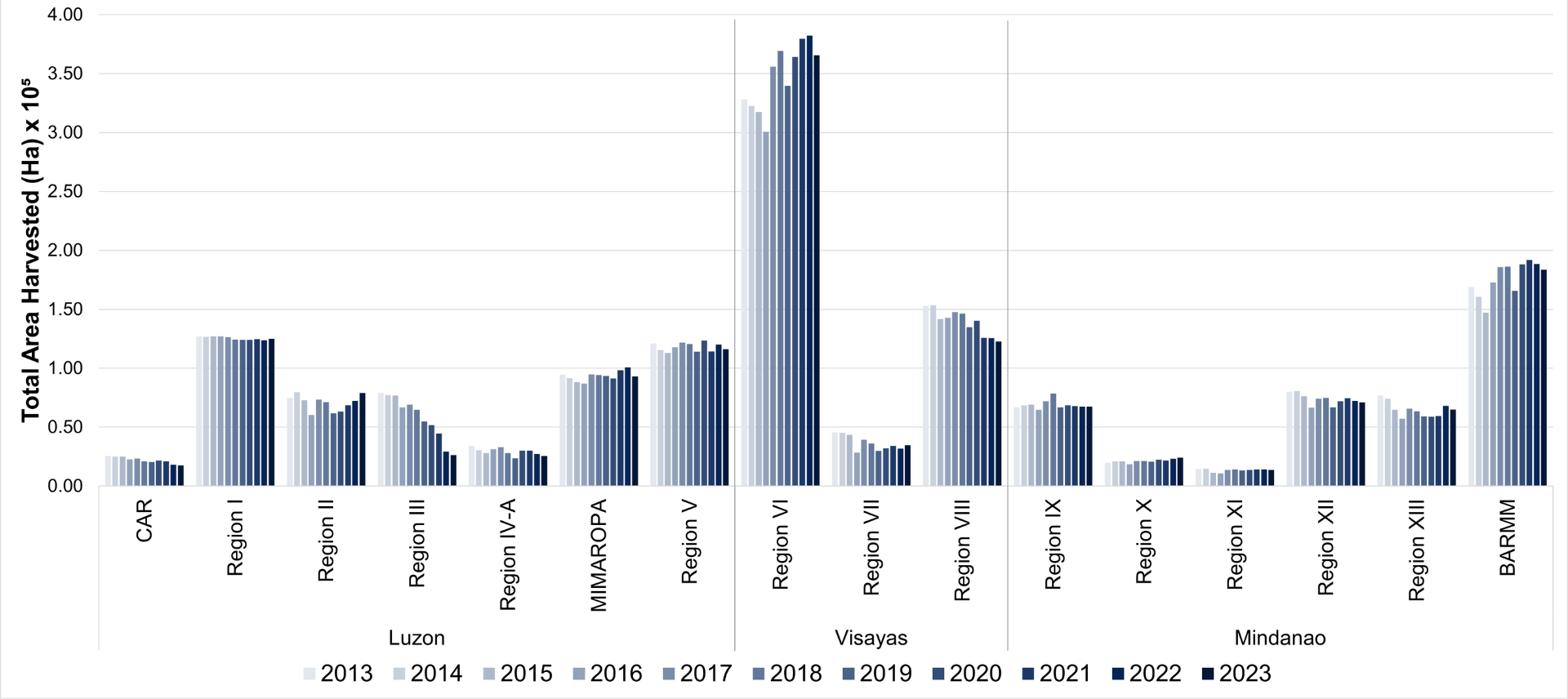

Supplement: S12 Fig — Figure was produced using data from the Philippine Statistics Authority [21]. (TIF) [file pone.0335344.s012.tif]

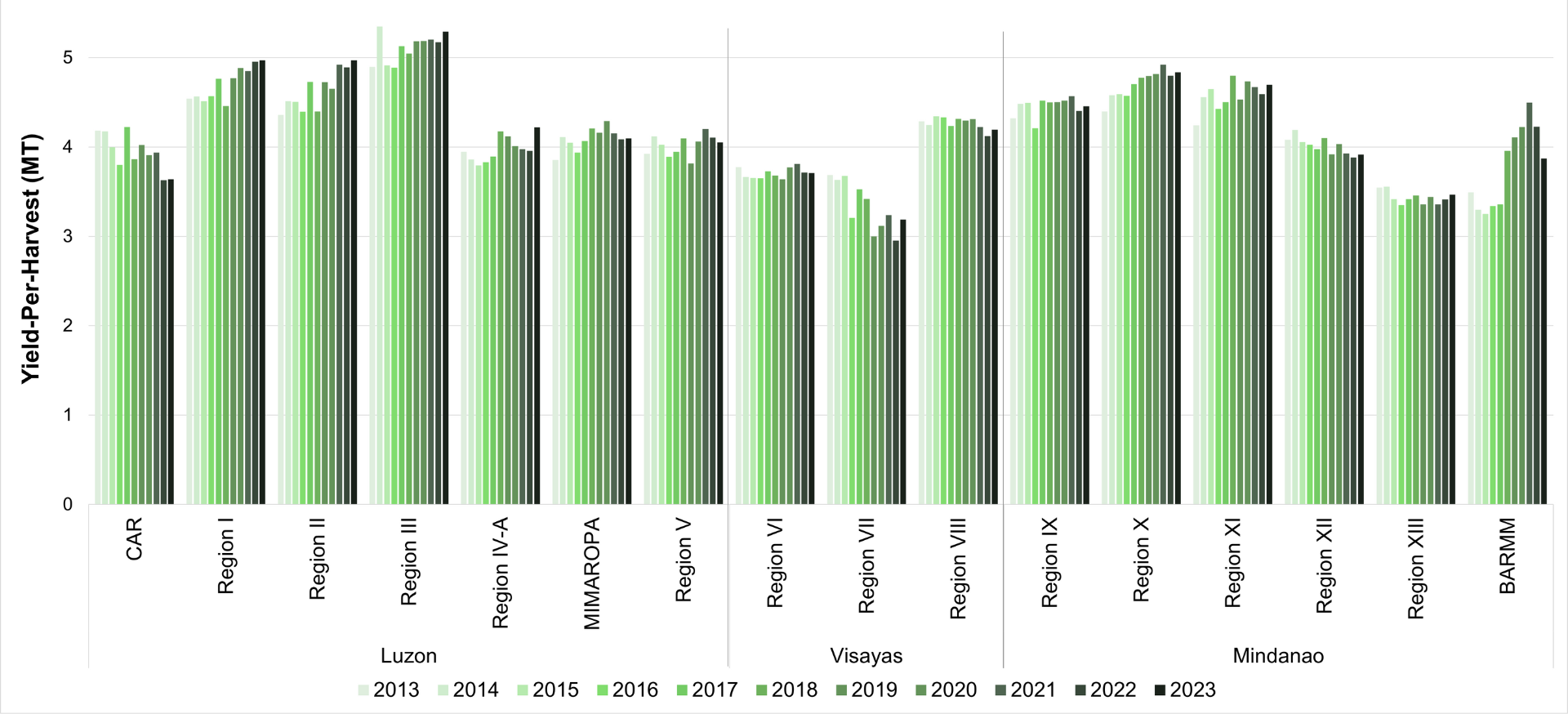

Supplement: S13 Fig — Figure was produced using data from the Philippine Statistics Authority [19,21]. (TIF) [file pone.0335344.s013.tif]

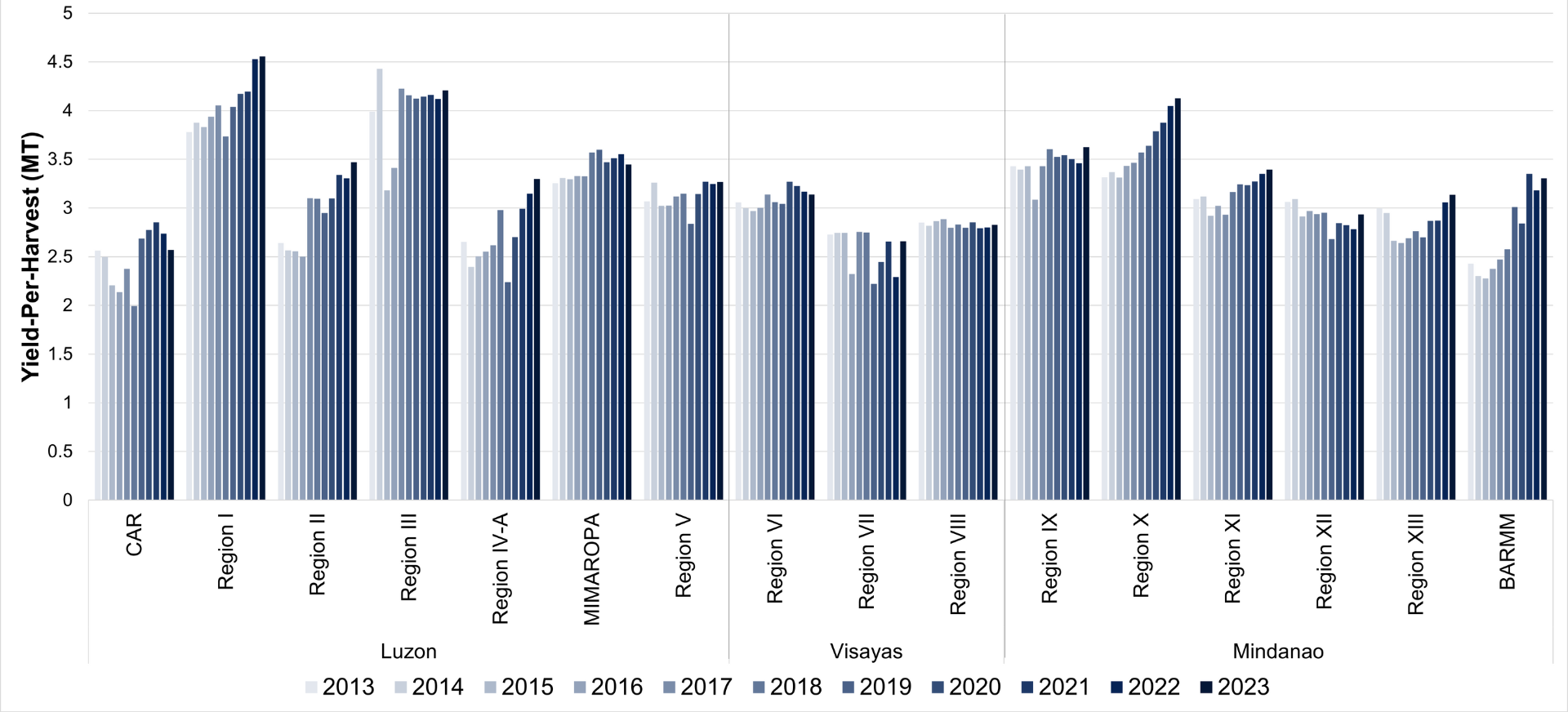

Supplement: S14 Fig — Figure was produced using data from the Philippine Statistics Authority [19,21]. (TIF) [file pone.0335344.s014.tif]
